# FEM analysis in excellent cushion characteristic of ostrich (*Struthio camelus*) toe pads

**DOI:** 10.1371/journal.pone.0216141

**Published:** 2019-05-22

**Authors:** Rui Zhang, Lei Ling, Dianlei Han, Haitao Wang, Guolong Yu, Lei Jiang, Dong Li, Zhiyong Chang

**Affiliations:** 1 Key Laboratory of Bionic Engineering, Ministry of Education, Jilin University, Changchun, PR China; 2 China North Vehicle Research Institute, Beijing, PR China; 3 Beijing Institute of Spacecraft Environment Engineering, Beijing, PR China; University of Illinois at Urbana-Champaign, UNITED STATES

## Abstract

African ostrich (*Struthio camelus*) is the largest and fastest extent bipedal animal. The ostrich mainly relies on the 3rd toe to support the entire body under high-speed motion. The short and severe impact concentrated on the limited area would produce tremendous momentary internal stress and strain, which may contribute to the phalanges disloaction, soft tissue damage and the like. The vibration and excessive negative acceleration caused by the ground reaction force also affect the stability of the touching process. Therefore, ostrich toe pads are required to have excellent cushion characteristics. However, current studies often explains the cushion properties by analyzing the macro-microscopic structure of the pad organism, and there is a paucity of research on its biomechanical behaviour. Consequently, from the perspective of multi-layer structure and biomaterial assembly, this study aims to explain the biomechanical characteristics of the ostrich toe pads by FEM (Finite Element Method) analysis. Based on results, we deem that the ostrich toe pad could absorb energy and reduce vibration effectively. Firstly, the multi-layer structure of the pads make the stress and strain decay from outside to inside. Secondly, the minimal response frequency of the pad is 164.22 Hz, making it effectively avoid resonance phenomenon. Finally, the composite material model has the best performance in decreasing the negative acceleration peak value, the impact force peak value and the maximal equivalent stress value at velocities of 0.669 m/s and 1.339 m/s. These results help to further understand the buffer mechanism of the ostrich toe pad, and also have important inter-species reference value for the pathogenesis of human foot soft tissue injury.

## Introduction

After long-term evolution, animals, with large weight or excellent athletic ability, often have a unique foot pad structure to deal with the damage caused by ground reaction to the soft tissue of the foot during exercise. The African elephant foot pads consist of bundle-like or fibrous connective tissue, and form a small space in the middle, which was filled with adipose tissue. This structure can support weight, absorb and disperse mechanical shocks [1]. The foot pads of the horse consist of dense connective tissue, mucoid tissue, cartilage and a small amount of single-room adipose tissue [2]. In cattle, the foot pad consists of resilient loose connective tissue containing varying amounts of associated soft fat enclosed in the envelope of collagen fibers. This cushion supports a greater proportion of body weight [3]. Histological analysis of the human fat pads indicated that a meshwork of fibroelastic septae arranged in a closed-cell configuration, which played a major buffering role [4]. The ultra-microscopic structure of the toe pad indicates that there are two kinds of cells, the typical signet ring and the diffused form cells. Elastic fibers can reshape fat cells, which could absorb shock and store energy so as to avoid excessive local stress on the ostrich phalanges [5].

A large number of literature often expound the animal cushion properties of footpads from macro-microscopic structures. However, these literatures do not explain the cushion mechanism of the footpad from biomechanical aspects. The cushioning properties of the footpad tend to focus on the soft tissue protection and the stability.

There are many factors that cause damage to the soft tissue of the foot, mainly including increased stretching of the tissue, excessive stress [6]. Davis B L. gives the value of the minimum friction coefficient ((μ_Rmin_) required to prevent slip. If the value is greater than the actual coefficient of friction (μ_A_), local deformation may occur, which may result in three skin ulcers [7]. Miao H investigated the relationship between structural features and cushion functions by analyzing the Mises stresses distribution and deformation of each layer [8]. Diabetes could stiffen soft tissue, thereby weakening its ability to disperse ground loads. Amit Gefen found that compared with normal, simulation model revealed significant tension stress concentrations, and the average internal soft tissue stress of the tibia increased by 82–307% [9].

The stability of the foot pad is mainly to reduce the vibration and the rebound force. When paws with elastic pads struck the ground, they might be set into oscillation, causing a 'chattering' phenomenon. A model with viscoelastic properties could reduce vibration effectively [10]. Zhang assumes that the cat's claw is a viscoelastic model, and the simulation test shows that the model can not only reduce the initial impact, but also effectively prevented the foot instantaneously rebounding from the ground [11].

The ostrich is the extant largest bipedal ratite, living in the desert and wasteland. Additionally, digitigrades generally move more quickly than other animals. Thus, their foot pads may be subjected to larger transient ground reaction forces, as high as six times their body weight [12–15]. Zhang et al. and Schaller et al studied the ostrich plantar pressure distribution during the ostrich walked or ran gait through the dynamic pressure plate. The results showed that the 4th toe mainly played an auxiliary balance, and the plantar load was concentrated beneth the 3rd toe [16,17].

In this paper, from the aspects of energy absorption and vibration reduction, FEM analysis was used to explain why ostrich toe pad could protect the plantar tissue and maintain stability. The mechanical properties of the skin, fascia and toe cushion of the toe pad were measured respectively. Four contrast models were used to illustrate the effects of material assembly and multilayer structure on buffering capacity.

## Materials and methods

### Biomaterial parameter measurement

#### Sample

This study was approved by the Institutional Animal Care and Use Committee (IACUC, protocol number: 20140706) of Jilin University, PR China. Two healthy sub-adult ostriches with an average age of 8 months were selected from a large ostrich farm in Ji’an breeder, Jilin Province, PR China. The average mass and height of the two ostriches were 83.5 kg and 2.10 m, respectively. The samples were without any form of surgical treatment or invasive physical manipulation. After the ostrich slaughter, the right foot was amputated and subjected to anti-dehydration treatment. Samples are stored at -20°C for a period of no more than one day before processing.

Before data acquisition, samples were thawed at room temperature. One of the samples was used for Magnetic Resonance Imaging (MRI) scan, and the other for biomaterial testing. The MRI scanning was performed on the sample to obtain the precise distribution of the structures, using a Discovery MR750 3.0T MRI apparatus. The generated image was stored in DICOM format. Under the guidance of previous studies [18], the skin, fascia and toe cushion of the 3rd toe pad were separated from the phalanges.

#### Hardness

In this study, LX-A Shore hardness tester was used to test the hardness of the ostrich skin, fascia and toe cushion. During the test, the components were placed flat on the glass plate, and the hardness tester was vertically pressed into the surface of the test piece. After the pressure pin was stable, the data was read. Five sample points with a spacing of not less than 5 mm were selected for each test.

#### Elastic modulus

The elastic modulus of the the skin and fascia were measured by UH4503, a universal testing machine of Shanghai You hong Measurement & Control Technology Co., Ltd. The maximum test force of this tester is 500 N, and the minimum displacement resolution is 0.001 mm. It is mainly used for the tensile test of rubber and other similar materials. Before the test, the piece was firstly made. The skin is an anisotropic material, so the direction of loading will have an influence on the determination of the mechanical properties. Therefore, the loading direction was parallel to the direction of the skin texture for the unification of mechanical properties. The GB/T2941 cutter and slicer were used to process the the stratified epithelium layer into dumbbell-shaped standard specimens.

The sandpaper was wrapped in the clamping part to protect the tested soft tissue specimen and increase the friction. Throughout the test process, the result of severe slippage was discarded, and the same to the fractured specimens in both ends rather than in the narrow region. After clamping and fixing, the UH4503 was used to tensile samples at a constant speed (20 mm/min) until it breaks. In order to ensure the accuracy and reliability of the test data, five valid repeated tensile tests were performed.

The toe cushion has a large viscosity. It is difficult for a traditional mechanical test method to accurately measure its elastic modulus. Therefore, a nano indenter was used for the test. The indenter was pressed into the surface of the measured object and loaded at a certain rate. The load-displacement curve of the material was obtained by measuring the load acting on the indenter and the depth of the surface pressed into the sample.

#### Density

Before test, the piece was made firstly. The the subcutaneous layer tissue was hard to cut due to its low hardness. Therefore, it was placed in a freezer at -20°C for freezing before cutting. A vernier caliper was used to measure the size of the specimen, and mass weighed was by electronic scale. Sample size and quality are shown in the Table 1.

**Table 1:** Sample size and quality.^a^

Toe pad component	Length (mm)	Width (mm)	Thickness (mm)	Quality (g)
Skin	30.45±0.29	7.24±0.14	1.87±0.06	0.33±0.02
Fascia	35.23±0.21	4.99±0.04	1.71±0.03	0.23±0.01
Toe cushion			7.57±0.23	0.35±0.02

^a^ Values are displayed as means ± S.D.

### Finite element model

This experiment was based on 3D MRI scan and obtained images of various parts of an ostrich toe pad as raw data for modeling. Then, using the reverse engineering software Geomagic Studio, a 3D model was generated for Abaqus simulation analysis [19]. The toe pad was placed between the upper and lower plates to simulate the interaction with the bone and the ground. The upper plate was used to imitate the bone and the lower plate was used to imitate the ground. The properties used in the toe pad model were measured according to the biomaterial test. Since the toe pad had a large difference in stiffness from the bone and the ground, the deformation occurring in the finite element analysis was minimal. Therefore, in order to save computational resources and increase the speed of the model, the upper and lower plates were defined as rigid bodies. The reference point (RP) was the load and force control point, which was located in the middle of the upper surface of the cube, and the point was coupled to the peripheral nodes of the yellow area.

#### Pre-processing grid division

Prior to finite element analysis, free meshing was used to mesh the toe model. After meshing, the skin model got 22006 units, the fascia model 21085 units, and thecushion model 15668 units in total.

#### Static pressure analysis model

**Fig 1:**
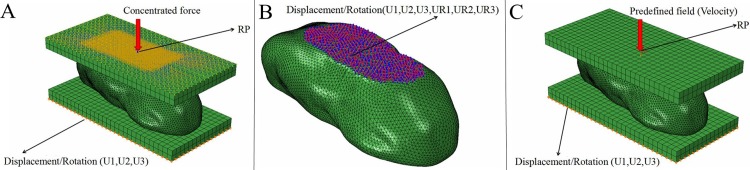
Model setting. A. Static analysis model. B. Modal analysis model. C. Dynamic analysis model.

The weight of the sample was about 80 kg. Assuming that the weight was evenly distributed on the two feet, that is, the load of one foot was about 400 N. The upper was applied a vertical downward 400N concentrated force load at the RP, limited to 5 degrees of freedom except the vertical direction, and the bottom was completely fixed. The load imitated the force exerted on the toe pad by the ostrich weight weight at rest. The model drawing is shown in Fig 1A.

#### Modal analysis model

Lanczos method was used to analyze the cushioning characteristics of ostrich paw pads in this study. The toe pad vibration was regarded as a multiple degrees of freedom undamped vibration system.

[M]{Ψ}+[K]{x}={0} (1)

In general, the undamped vibration of the elastomer was the result of superposition of some simple harmonic vibrations. Therefore,the Eq (1) could be obtained.

{x}=Xsin(ωt+ε) (2)

$$\left\{\Psi \}=-\omega^{2}X\;sin\;(\omega t+\alpha \right)$$ (3)

According to Formulas (2) and (3), the solution of Formula (4) could be got.

$$[K]\{x\}-\omega^{2}[M]\{x\}=0$$ (4)

In the process of free vibration, the amplitude of each node, {x}, of the structure was not zero. Therefore the necessary and sufficient condition for the non-zero solution of the homogeneous equations was that the coefficient determinant was zero.

$$|[K]-\omega^{2}[M]|=0$$ (5)

Finally, the vibration mode frequency values of each order could be obtained.

In the process of modal analysis, no translation,rotation constraints and loading conditions were applied to the ostrich toe pad. Only the nodes at the bottom of the toe pad were collectively set and a fixed constraint was imposed on the it. That is, The model was subjected to a 30-order modal analysis in a free state to obtain the model frequency and mode of vibration [20]. Modal analysis was based on the theory of linear vibration and could ignore the effects of damping. Based on the above analysis, we can obtain the natural frequency and mode of vibration of the paw pad model. The finite element model is shown in Fig 1B.

#### Dynamic analysis model

The dynamic analysis imposed fixed constraints on the lower plate, and set surface contact with the toe pad model. The mass of the upper plate was set to 40 kg, and an initial impact speed was applied to it to simulate the impact of an ostrich foot. The analysis step time was set to 0.02 s, divided into 100 solving steps. The speed imitated the impact of bone on the toe pad during exercise. The established model is shown in Fig 1C.

At the same time, the dynamic simulation analyzed the influence of the material properties and multi-layer structure of the ostrich toe pad on the cushion performance. Based on this, four models were established and the corresponding simulation calculations were performed in Abaqus.The distribution of the four models from the outer layer to the inner layer is as follows:

- Model 1: the skin layer, the fascia layer, the toe cushion layer
- Model 2: the skin layer, the fascia layer, the fascia layer
- Model 3: the skin layer, the toe cushion layer, the toe cushion layer
- Model 4: the skin layer, the fascia layer, none

**Fig 2:**
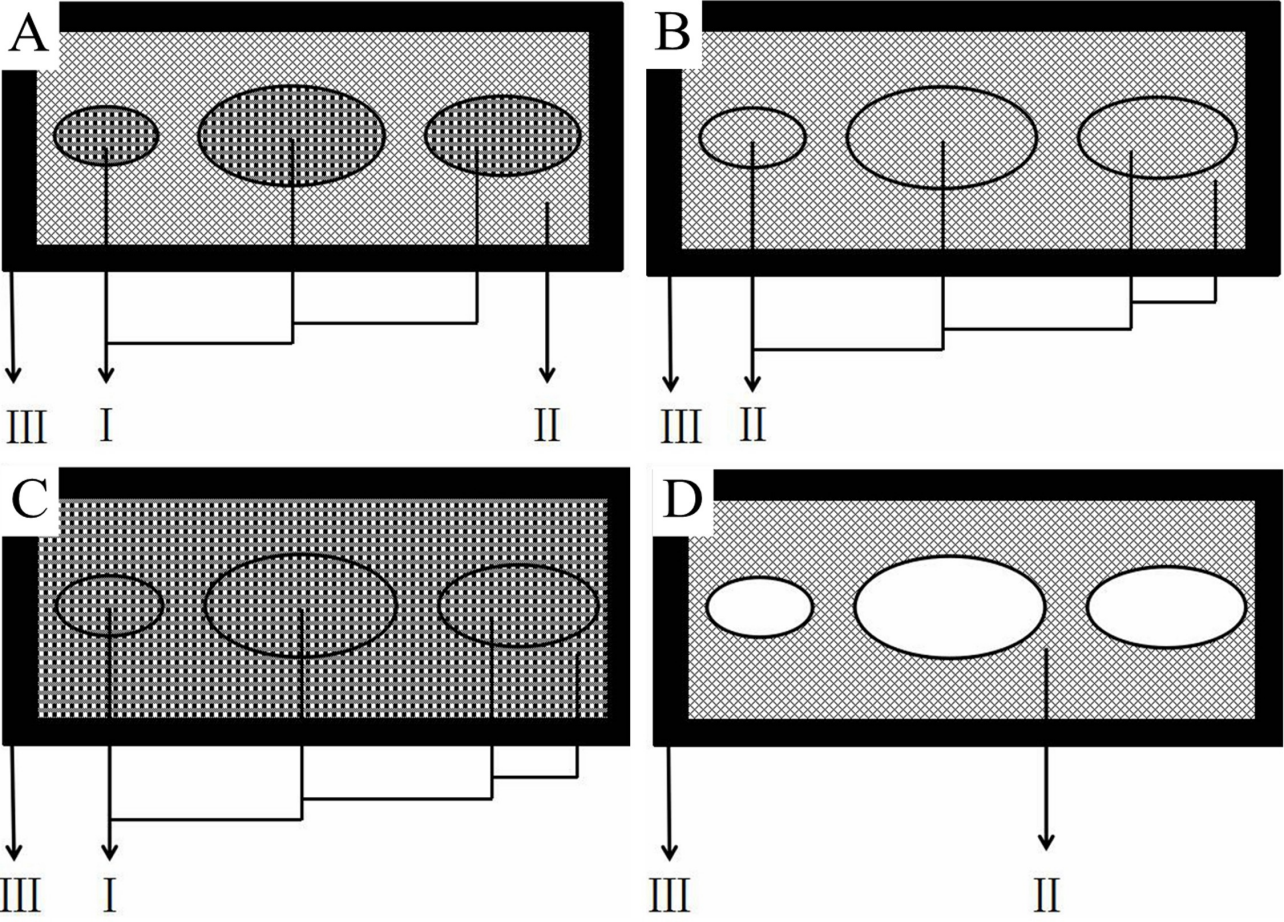
Schematic diagram of the distribution of the four model materials. A-D corresponds to the model 1–4, respectively.

Model 1 was the assembly according to the biological prototype, Model 2–4 were the control models respectively. To facilitate analysis, the toe cushion layer, the fascia layer, and the skin layer were named I, II, and III, respectively. A schematic diagram of the distribution of the four models is shown in Fig 2.

## Results

### Biomaterial parameters

**Fig 3:**
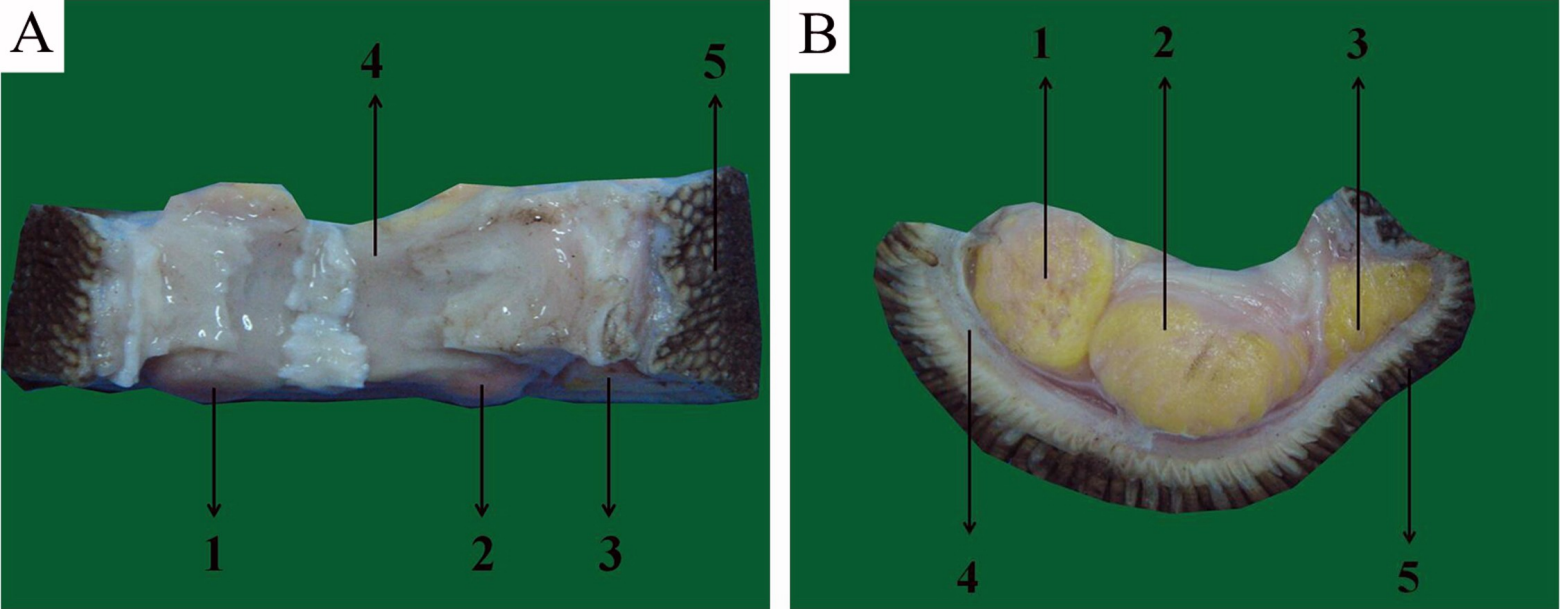
Results of the gross anatomy. A The structure of the ostrich toe pad, top view. B The structure of the ostrich toe pad, top view, lateral view.

By gross anatomy, the structure of the ostrich toe pad is shown in Fig 3. It could be seen that the paw pad was mainly composed of three layers. Three irregularly tubular, yellow toe cushion, are shown in 1, 2, and 3. The outer wrapped fascia is shown in 4, and the outermost skin is shown in 5.

Through the biomaterials test, the results of the three-layer structure of the ostrich toe pad are shown in Table 2.

**Table 2:** Results of biomaterials test.^a^

Toe pad component	Shore hardness	Elastic modulus (MPa)	density (g/mm^3^)
Skin	65.8±2.3	8.30±1.76	(80.7±3.15)×10^−5^
Fascia	41.9±1.9	6.38±1.37	(74.5.±1.57)×10^−5^
Toe cushion	31.8±1.9	3.77±2.33	(73.0±2.11)×10^−5^

^a^ Values are displayed as means ± S.D.

### Finite element analysis

#### Static pressure analysis

Static pressure analysis obtained the state of the ostrich toe pad during static standing. Under the static load of 400 N, stress, strain and displacement distribution of the model were analyzed. The calculation results were as following.

**Fig 4:**
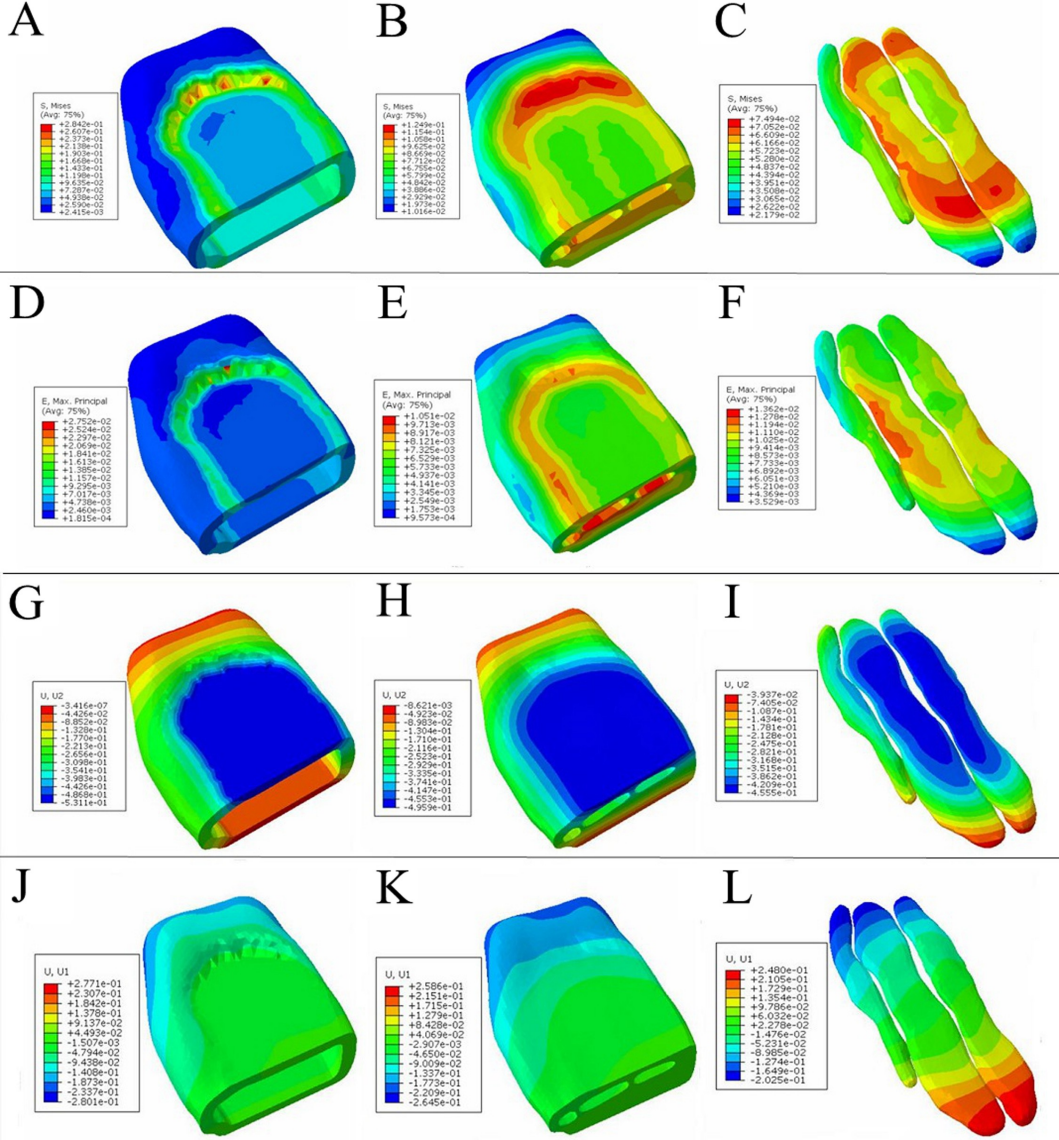
Static pressure cloud map of the ostrich toe pad model. A-C corresponds to the equivalent stress of skin, fascia, and toe cushion, respectively. D-F corresponds to the strain of skin, fascia, and toe cushion, respectively. G-I corresponds to the longitudinal displacement of skin, fascia, and toe cushion, respectively. J-L corresponds to the lateral displacement of skin, fascia, and toe cushion, respectively.

Under the static load compression state, the distribution of the equivalent stress distribution of the ostrich toe pad is shown in A-C of the Fig 4. It could be seen that the maximum equivalent stress of the skin was 0.28 MPa. The stress fluctuated between 0.07–0.28 MPa in the middle region, andthat was small in the surrounding area. The maximum equivalent stress of the fascia was at 0.12 MPa. The stress fluctuated between 0.05 and 0.12 MPa in the middle region, and varied from 0.01 to 0.05 MPa in the surrounding area. The maximum equivalent stress of the toe cushion was 0.07 MPa. The strain fluctuated between 0.04 to 0.07 MPa in the middle region, and that was small in the surrounding area.

The structural strain of each layer of the ostrich toe pad model is shown in D-F of the Fig 4. It can be seen that, the maximum strain of the skin was 0.028. The strain in the middle region fluctuated between 0.007 to 0.028, and that was small in the surrounding area. The maximum strain of the fascia was 0.011. The strain in the middle region was fluctuations ranged from 0.004 to 0.011, and that was small in the surrounding area. The maximum strain of the toe cushion is 0.014. The strain in the middle region ranged from 0.008 to 0.014, and in the surrounding area was from 0.004 to 0.008.

The vertical and lateral deformation displacements of the toe pad under the influence of 400N external load are shown in G-L of the Fig 4. According to the cloud map, the maximum of the skin in the longitudinal direction under external force was 0.53 mm, and in the lateral direction was 0.28 mm. The maximum deformation in the longitudinal direction of the fascia was 0.49 mm, and in the lateral direction was 0.26 mm. The maximum deformation of the toe cushion in the longitudinal direction was 0.46 mm, and that was 0.25 mm in the lateral direction.

#### Modal analysis

Through modal analysis, the natural frequency of the first 30 order of the ostrich toe pad and the participation coefficient of each degree of freedom are shown in Table 3. The natural frequency of an object is only related to its own properties such as material and mass properties. Through analyzing the participation coefficient, the motion intensity of the model in each direction of freedom could be obtained. That is, the motion mode of the analysis model was mainly based on translation or rotation [21].

**Table 3:** The modal natural frequencies of the first 30 stages of ostrich toe pads.

Modal order	natural frequency (Hz)	Participation coefficient of each degree of freedom					
		X-Translation	Y-Translation	Z-Translation	X-Rotation	Y-Rotation	Z-Rotation
1	164.22	-0.228	0.119	1.434	395.4	-119.8	71.23
4	273.30	0.226	1.141	-0.004	80.46	-13.27	46.91
7	392.89	-1.501	-0.049	-0.190	-64.43	117.8	472.6
10	508.79	-0.001	1.103	-0.143	28.15	13.07	106.3
13	658.94	-0.031	0.075	0.293	97.81	-27.20	15.30
16	700.22	-0.037	-0.013	0.098	31.93	-7.586	10.68
19	822.64	-0.084	-0.005	-0.021	-6.349	6.020	24.40
22	885.12	0.044	-0.003	-0.046	-14.03	5.527	-13.16
25	920.42	-0.137	-0.068	-0.175	-55.77	22.82	32.93
28	969.00	-0.035	0.269	-0.159	-29.83	14.79	37.09
30	997.38	-0.103	-0.131	-0.145	-51.89	18.92	17.46

From Table 3, we could see that the minimum response frequency of the ostrich toe pad was 164.22 Hz. When the external excitation frequency was lower than this value, the ostrich paw pad could maintain sufficient dynamic stiffness to prevent resonance. In addition, the participation coefficient values were on average about 1–2 orders of magnitude lower in the main translation direction than those in the rotation direction, namely, the translation characteristics were negligible relative to the rotation characteristics. The model exhibited a distinct rotational mode of vibration.

**Fig 5:**
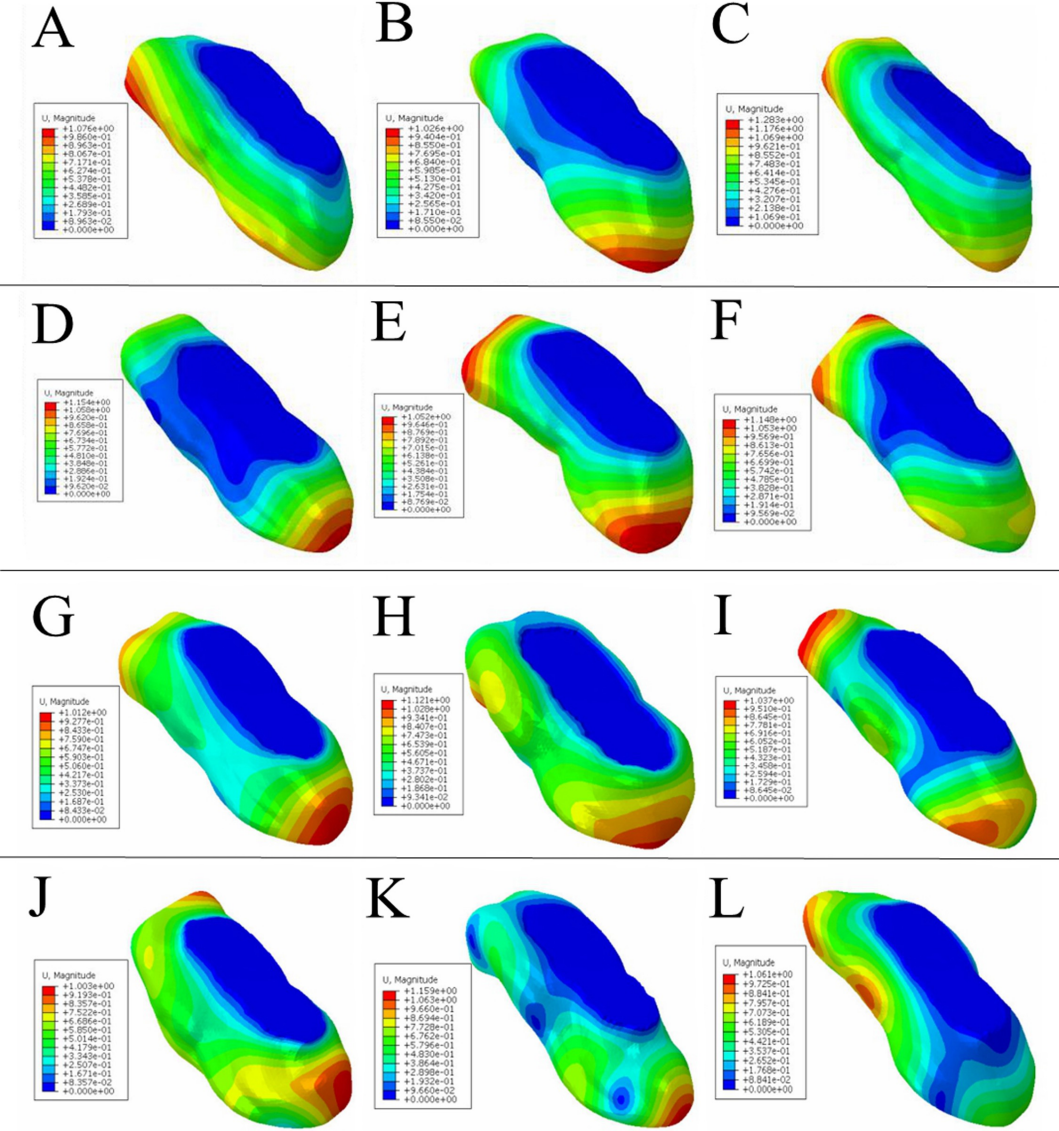
The modal analysis of the ostrich toe pad. A-L corresponds to the 1st-12th modal analysis, respectively.

The 12 order non-rigid body mode of vibration and the displacement vector of the toe pad surface are shown in Fig 5. It could be seen that the 2nd, 4th, 7th, 8th, and 11th order mode of vibrations mainly occurred at the front end of the ostrich paw pad, local deformation occurred at the front end, and those were small the middle and back end distortions. The 1st, 3rd, 6th and 12th order mode of vibrations mainly occurred at the rear end of the ostrich toe pad, with local deformation at the rear end, and the middle and front end deformations were small. The 5th, 9th, and 10th order mode of vibrations mainly occurred at both ends. There was local deformation at both ends, and the middle part was less deformed.

#### Dynamic analysis

**Fig 6:**
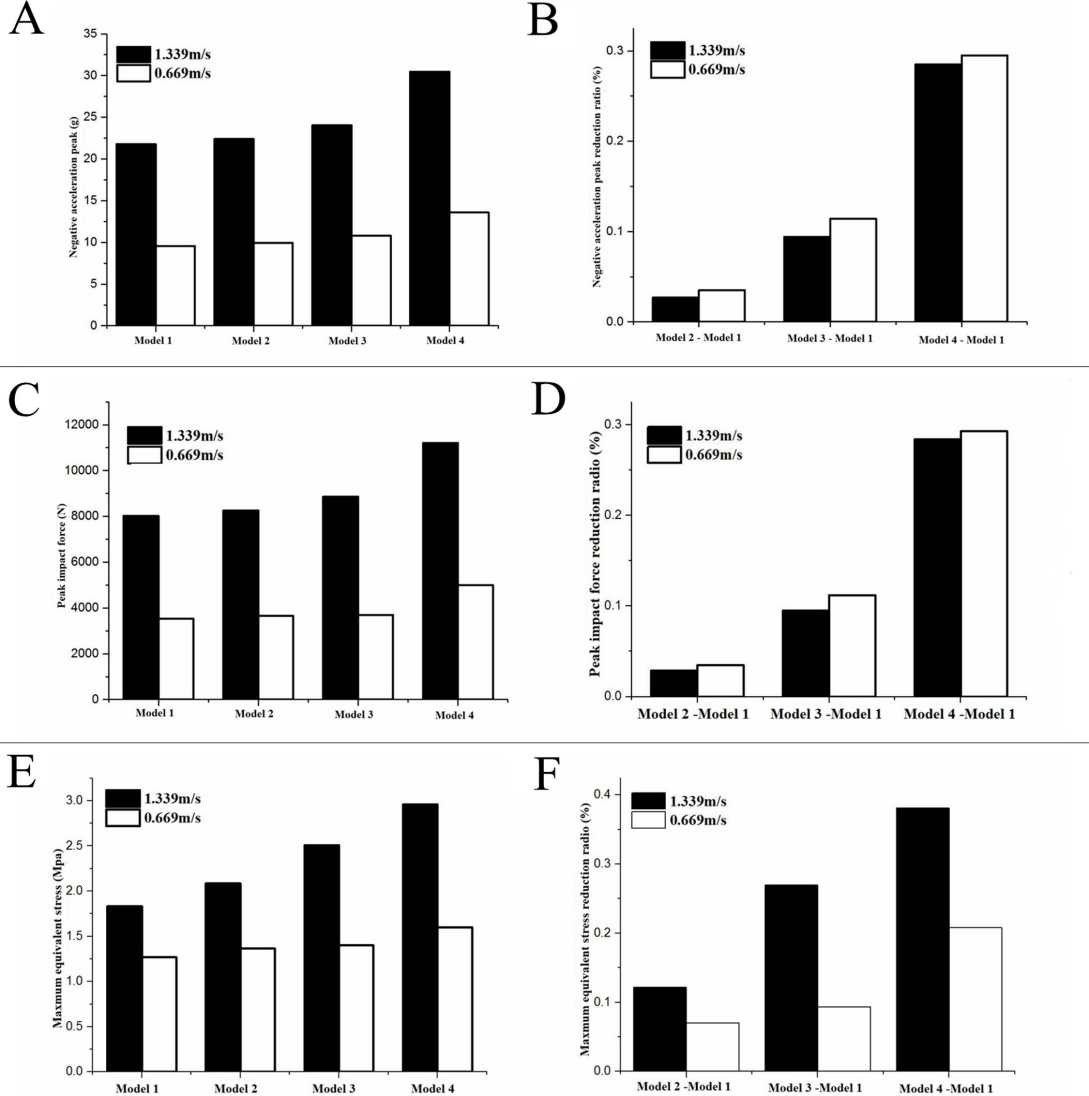
Comparison of peak impact forces, equivalent stress distribution and the maximum equivalent stress of the four models. A Negative acceleration peak. B Negative acceleration peak reduction ratio. C Peak impact force. D Peak impact force reduction ratio. E Maximum equivalent stress. F Maximum equivalent stress reduction ratio.

The peak of negative acceleration could be used as an important indicator of the buffering effect. Under the same impact conditions, the smaller the negative acceleration peak was, the better the buffering effect. Through the finite element simulation calculation, it could be seen that the peak value of negative acceleration of the four models reached 21.78g, 22.38g, 24.03g and 30.47g respectively at the impact velocity of 1.339 m/s. Compared with the other three models, the negative acceleration of model 1 decreased by 2.7%, 9.4%, and 28.5% respectively. Similarly, at the impact velocity of 0.669m/s, the negative acceleration peak of the four models reached 9.57g, 9.93g, 10.81g, and 13.58g respectively. Compared with the other three models, the peak acceleration of model 1 decreased by 3.6%, 11.4% and 29.5% respectively, as shown in Fig 6A and 6B.

The peak impact force was also an amount commonly used to describe the buffering effect. Under the same impact conditions, the smaller the peak impact force was, the better the buffering effect. By analysis, the peak impact force of the four models reached 8017.2 N, 8253.5 N, 8854.6 N, and 11199.4 N respectively at an impact velocity of 1.339m/s. Compared with the other three models, the impact force of model 1 The peak values decreased by 2.9%, 9.5%, and 28.4% respectively. Simultaneously, at the impact velocity of 0.669m/s, the impact peak of the four models reaches 3530.5 N, 3658.8 N, 3976.2 N, and 4996.6 N, respectively. Compared to the other three models, the peak impact of model 1 decreased by 3.5%, 11.2%, and 29.3%, respectively, as shown in Fig 6C and 6D.

**Fig 7:**
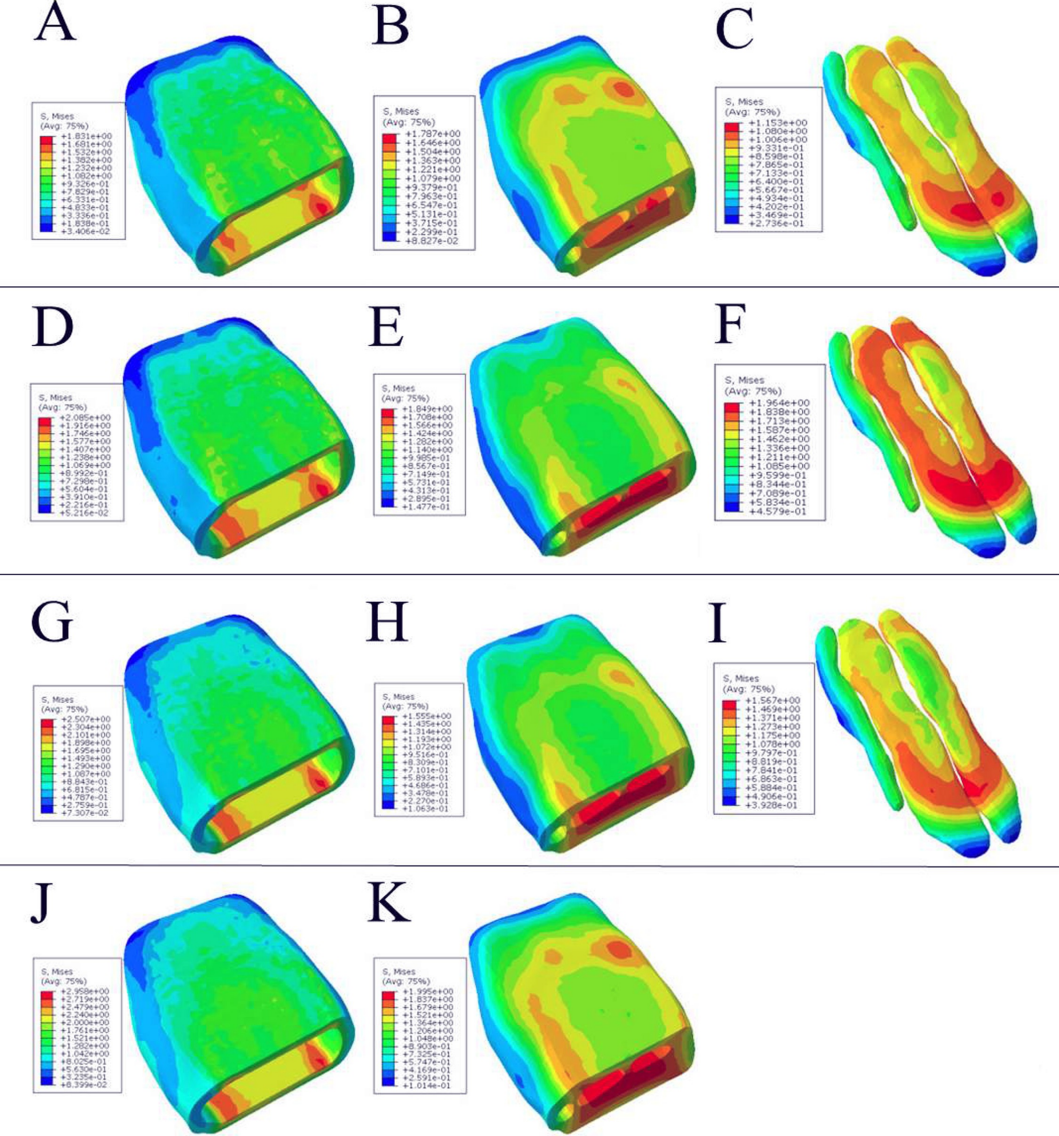
Equivalent stress distribution of four models. A-C corresponds to the skin, fascia, and toe cushion of model 1, respectively. D-F corresponds to the skin, fascia, and toe cushion of model 2, respectively. G-I corresponds to the skin, fascia, and toe pad of model 3, respectively. J-K corresponds to the skin and fascia of model 4, respectively.

After the finite element dynamic analysis, the maximum equivalent stress distribution cloud diagram for each layer of the four models at the impact velocity of 1.339 m/s was obtained, as shown in Fig 7. It could be seen that the maximum equivalent stresses (Von Mises stress) of the four model skin was 1.83 MPa, 2.09 MPa, 2.51 MPa, and 2.96 MPa, respectively. The maximum equivalent stress of the fascia was 1.79 MPa, 1.85 MPa, and 1.56 and 1.99 MPa, respectively. The maximum equivalent stress of the first three model the toe cushion was 1.15 MPa, 1.96 MPa, and 1.57 MPa, respectively.

By further analysis, it was compared that the maximum equivalent stress under the two impact conditions, the impact velocity 1.339 m/s and 0.669 m/s. Just as shown in Fig 6E and 6F, the maximum equivalent stress of the toe pad model showed an increasing trend with the impact velocity increases. Under the two conditions, compared with model 2, the maximum equivalent stress of model 1 decreased by 12.2% and 7.1%, respectively. Compared with model 3, the maximum equivalent stress of model 1 decreased by 26.93% and 9.4%, respectively. Compared with model 4, the maximum equivalent stress of Model 1 decreased by 38.1% and 20.8%, respectively.

## Discussion

This study conducted a gross dissection of the toe pad of the ostrich right foot and found that it consists of multi-layer structure, which were the skin, fascia and toe cushion, that is, the stratified epithelium layer, the intermediate dermis layer and the subcutaneous layers. SA El-Gendy et al. and Miao et al. also described similarly in previous studies [11,22]. Through biomaterial testing, we found that the three layers of material showed a tendency to soften from the outside to the inside. We speculate that this may be related to the different stress environments and functions between them. The skin and fascia were subjected to tremendous pad-ground wear, friction and impact during locomotion [23]. The toe cushion was the softest tissue material, filled with a large number of non-movable fat cells. This could be seen as a large hydrostatic system, which was able to absorb energy effectively [24]. This cushion characteristic about the differential biomaterial properties and multilayer structure is worthy of our quantitative analysis by FEM.

### Static pressure analysis

Due to material properties and spatial differences, the layers of the toe pad exhibit different characteristics in the static pressure simulation. Adult ostriches weigh between 80 and 200 kg. The load is mainly concentrated on the 3rd toe, with an area of 90 cm^2^ [15,16,25]. Stress distribution was relatively uniform and there was no stress concentration area. Standing for a long time would not cause local tissue ulcers caused by stress concentration [9]. From the stress, strain and displacement of the skin, fascia and toe cushion, it could be seen that the value of the toe pad decreases from the outside to the inside in order. The multi-layer structure can effectively reduce the damage of the outer load to the tissue wrapped by the toe pad. The maximum displacement was 0.53 mm and the maximum strain was 0.028 occurring in the skin layer. External loads did not cause excessive deformation of the soft tissue, resulting in soft tissue strain.

### Modal analysis

Xu found that 4–12.5 Hz is the most sensitive frequency range of organisms [26]. Through modal analysis of the ostrich toe pad model, it could be concluded that the lowest response frequency was 164.22 Hz, so it could be inferred that it was difficult for external stimuli to cause toe pads vibration, which suggested that the structure had good dynamic stiffness to avoid resonance. Studies about the modal analysis of humans and woodpeckers also found that the modals of vibration were mainly rotation-based, and the translation motion was relatively small [21,27]. It could be speculated that the rotational motion of the organism's body structure dominates. According to the analysis, the value of the translation participation coefficient was about 1–2 orders of magnitude lower than the rotation. Due to the similar external load excitation, the amplitude of the vibration would greatly increased. Therefore, the toe pad tissue of the ostrich can effectively relieve the translation motion, but rotation injury prevention ability was weak. However, from the modal analysis of the 12 non-rigid vibrational cloud patterns, it could be concluded that the toe pad was mainly bent at both ends, and the deformation of the middle was smaller. The soft tissue was mainly concentrated in the middle, so the damage due to the rotation would be greatly reduced.

### Dynamic analysis

Increased biomechanical properties of plantar soft tissue under the first metatarsal head in diabetic patients may result in higher plantar pressure peak and plantar pressure gradient [28]. In order to study the influence of material properties to cushion characteristics, four comparative models were designed. According to the way the materials assembled, they could be understood as that: model 1 was hard-intermediate-soft combination, model 2 was hard-intermediate-intermediate combination, model 3 was hard-soft-soft combination, and model 4 was hard-soft-none combination. It could be clearly observed that the three-layer material structure had a better gradient weakening effect on the external impact than the two-layer. By comparing Model 2 with Model 3, the peak impact force and average stress of the external structure would increase significantly when the inner layer was softer. Model 4 had the least cushion fuction due to the discontinuity of the structure. Model 3 has a larger negative acceleration peak than Model 2. That is, softer material properties did not imply better cushion abilities. Because this would seriously affect the stability of the touchdown process [10]. The greater speed produced the greater impact, but the layers of the toe pad had a similar tendency to slow down the impact.

## Conclusion

Through gross anatomy and biomaterial testing, the study found that the ostrich toe pad has a multi-layer structure, and their materials show a tendency soften to the inside. Though static pressure analysis, it could be concluded that the stress distribution of the toe pad is relatively even during static standing, and there was no stress concentration. In addition, the stress, strain and displacement of the three-layer structure were gradually attenuated from the outside to the inside. The toe pad was effective in dispersing the load and without excessive deformation. By modal analysis, it can hardly cause response under external stimuli, and effectively avoid resonance phenomenon. The pads could alleviate the translation movement, but the ability to prevent rotational injury is weak. However, the toe pad was mainly bent at both ends, and the deformation of the middle was smaller. The soft tissue was mainly concentrated in the middle, so the damage due to the rotation would be greatly reduced. Based on the dynamic impact analysis, the three-layer material assembly model has the best cushion performance. If the internal material is too soft, the maximum impact force and stress level of the external structure will be significantly improved, and the stability will be deteriorated. Therefore, the footpad should have a moderate hardness to maintain the stability of the touchdown process. At different impact speeds, the layers of the toe pad had a similar cushion tendency. Results of this study would facilitate bionic structure design and provide an inter-species reference for the pathogenesis of human foot soft tissue injury.

## Supporting information

S1 Fig Comparison of peak impact forces, equivalent stress distribution and the maximum equivalent stress of the four models. A Negative acceleration peak. B Negative acceleration peak reduction ratio. C Peak impact force. D Peak impact force reduction ratio. E Maximum equivalent stress. F Maximum equivalent stress reduction ratio.
(XLS)

S1 Table The modal natural frequencies of the first 30 stages of ostrich toe pads. (XLS)
